# A Relational Workforce Capacity Approach to Trauma-Informed Care Implementation: Staff Rejection Sensitivity as a Potential Barrier to Organizational Attachment

**DOI:** 10.3390/bs13080652

**Published:** 2023-08-04

**Authors:** Tareq Hardan, Emily A. Bosk, Alicia Mendez, Abigail Williams-Butler, Fabrys Julien, Michael J. MacKenzie

**Affiliations:** 1 School of Social Work, McGill University, Montreal, QC H3A 1B9, Canada; 2 School of Social Work, Rutgers University, New Brunswick, NJ 08901, USA

**Keywords:** organizational attachment, intent-to-turnover, rejection sensitivity, Trauma-Informed Care (TIC), Attachment, Regulation and Competence (ARC), implementation science

## Abstract

This study explores the relationship between staff rejection sensitivity (a psychological concept grounded in histories of loss and trauma) and organizational attachment among mental health agencies transitioning to Trauma-Informed Care (TIC), which is currently outside the focus of most research. Specifically, this study examines: (1) whether staff rejection sensitivity predicts organizational attachment; (2) whether staff turnover intentions account for the association between rejection sensitivity and organizational attachment; and (3) whether those associations hold once taking into account staff demographic factors (gender, race and ethnicity, education, and income)? Around 180 frontline workers in three Northeastern U.S. mental health agencies responded to surveys collected between 2016 and 2019 using the organizational attachment, rejection sensitivity and turnover intention measures, and their previous TIC training experience. Rejection sensitivity was significantly associated with organizational attachment (β = −0.39, *p* < 0.001), accounting for 6% of its variance in organizational attachment. The relationship between these variables retained significance, and staff education significantly predicted organizational attachment, with higher education predicting lower levels of organizational attachment (β = −0.15, *p* < 0.05), accounting for 22% of its variance. This study concludes that TIC transitioning mental health agencies’ staff with a higher rejection sensitivity are more likely to express lower organizational attachment and higher intent-to-turnover.

## 1. Introduction

Trauma-Informed Care (TIC) refers broadly to programmatic, organizational, or systemic interventions designed to respond to trauma [1]. Due to the pervasiveness of trauma exposure and trauma symptoms among clients receiving mental health services, the Substance Abuse and Mental Health Services Administration (SAMSHA) in 2014 proposed that TIC implement interventions in a way that “actively resist re-traumatization.” (p. 9). Clients receiving mental health services require stable and engaged relationships with their providers to avoid the re-traumatization of clients that can occur when there is a discontinuity in their clinical team. Annual turnover rates in child welfare-serving agencies range between 14 and 22% [2], raising concerns about the ability of organizations to deliver on the promise of TIC.

In this context, it is critical to consider whether the additional emotional and task demands that accompany transitioning to TIC and shifting away from behavioral-based interventions may amplify already high turnover rates among mental health service staff. Little is known, however, about how mental health service staff’s organizational attachment informs turnover intentions in the context of the additional emotional demands required by TIC. To date, there has been no research investigating these relationships. This study seeks to fill these gaps by providing empirical data about the link between staff’s organizational attachment and intent-to-turnover in the context of the transition to TIC at three mental health agencies. Within these TIC transitioning agencies, the relational approach is prioritized, and staff relationships with their clients and supervisors require reshaping. Hence, understanding personal and organizational relational and affective factors for staff and not just clients is critical for successful TIC implementation.

### 1.1. Relational Safety as a Primary Component of TIC

In conjunction with relational safety, SAMSHA’s Trauma and Justice Strategic Initiative (2014) has identified “transparency, peer support, collaboration and mutuality, empowerment, voice, and choice, cultural, historical, and gender issues” (p. 10, [3]) as the core components of TIC. Despite the ubiquitous recognition of these principles, their implementation remains a challenge [4]. Relationships between clients and staff can create a pathway for recovery, creating healing trajectories by providing corrective emotional experiences [5]. The creation of relational safety can re-establish feelings of internal and external safety, which are often disrupted in traumatic experiences [6,7]. Effective intervention requires TIC service providers to foster a supportive relationship with their clients. Often, mental health providers must establish relational safety as they navigate multiple relational challenges from clients for whom emotional and behavioral dysregulation is a symptom of complex trauma [8]. Doing so requires mental health providers to continually regulate their emotional responses to their clients’ affective and behavioral dysregulation. Given that establishing relational safety is critical to effective TIC implementation, it is important to consider how frontline staff at mental health agencies’ relational characteristics influence TIC implementation.

### 1.2. Rejection Sensitivity

Staff’s relational styles can influence workplace interactions [9]. Insecure attachment styles manifest themselves in adults who expect rejection in interpersonal relationships and social settings [10,11]. Specifically, rejection sensitivity refers to the “intense dejection following [the] perceived rejection” (p. 232, [12]). Rejection sensitivity results in intense responses from insecurely attached individuals who expect rejection in interpersonal relationships and social settings [11]. A maladaptive response to a healthy social environment, rejection sensitivity is best conceptualized as a cognitive, affective, and behavioral “defensive motivational system”(p. 149, [13]) that shapes how people interpret and respond to the world around them. Individuals with high levels of rejection sensitivity are more likely to read ambiguous social interactions negatively and personally. When people interpret social interactions through the lens of rejection, negative feedback loops can occur, with angry or withdrawn responses guiding future interactions that in turn elicit and create the conditions for the initial (mis)perceived rejection. In mental health service settings, staff with higher levels of rejection sensitivity may, without proper organizational support, interpret clients’ symptoms of complex trauma personally. When this occurs, there is a higher likelihood that staff will respond to relational challenges from clients within a defensive motivational framework that can create a relational impingement on the therapeutic working alliance and the well-being of the staff member in that work setting. 

Rejection sensitivity has traditionally been studied in the context of interpersonal trajectories and relationships. Studies demonstrate that repeated rejection experiences can contribute to the coalescence of anxious rejection expectations that are defensively activated to protect against potential threats [11,14]. More limited research has begun to establish that rejection sensitivity influences work performance [15] and predicts burnout [16], even in settings that are not very relationally demanding. As a case in point, a higher rejection sensitivity for tenured and tenure-track business school faculty lowered their publishing efforts and publications’ quality [17]. In a more relationally demanding profession (teaching), another study found a 119% increase in the risk of burnout for university faculty with higher levels of rejection sensitivity after following them for 21 months [16]. In the workplace, higher rejection sensitivity may make people less likely to seek workplace support [18]. Moreover, adults with high rejection sensitivity also show a unique vulnerability to having their goal-directed activity disrupted when they perceive a social threat in the workplace environment, resulting in slower performance and attentional avoidance [19], affecting work performance [16]. Whether one expresses a higher sensitivity to rejection through withdrawal or anger, these ineffective management strategies often result in people having unfavorable views of themselves [20], which can likely negatively impact both relationships with colleagues and clients.

Newer research has begun to establish the link between staff rejection sensitivity and the implementation of TIC programs in mental health settings [9,21]. Specifically, Bosk et al. (2020) found that frontline staff with higher rejection sensitivity were less likely to be open to adopting TIC interventions in mental health agencies. Further, frontline staff were more likely to express an intent to increase turnover in mental health agencies transitioning to TIC. This line of work suggests that personal factors, specifically those related to rejection sensitivity, interact with work-related and organizational factors to inform staff’s likelihood of staying/leaving their organization, which in turn can influence TIC implementation. 

### 1.3. Turnover Intentions

Understanding the factors contributing to workforce retention is critical to effectively implementing TIC. Improving the retention of frontline staff supports relational safety by increasing provider continuity for clients. In addition to the relational implications of frequent staff turnover, when a high number of staff leave, an organization implementing new evidence-based models faces serious challenges in sustaining these programs. Training and coaching in specific TIC modalities such as Attachment, Regulation and Competency Model, Trauma-Focused Cognitive Behavioral Therapy, Child-Parent Psychotherapy (CPP), Positive Parenting Program (Triple P), or the Incredible Years involve a high cost and time investment for these organizations. High rates of staff turnover may make it difficult to train new staff in these interventions if the initial training commitments or staff time devoted to learning this modality have already ended. Staff turnover is therefore likely to be a significant and underexamined threat to the successful long-term implementation of evidence-based TIC interventions, all of which require intensive training and consultation. Identifying the factors that may motivate staff to leave requires close attention to improve TIC implementation. 

Kim and Kao’s (2014) meta-analysis of turnover intention among child welfare workers revealed four multileveled and relational factors influencing workforce intentions to leave: (1) staff job satisfaction and organizational commitment; (2) staff demographic characteristics such as age, gender, and education; (3) decision-making, stress, and burnout; and (4) organizational environment and support. Findings indicated that the staff’s lack of organizational commitment, a negative work environment, and perceived lack of organizational support strongly predicted the staff’s intent-to-turnover [22]. Additionally, organizational culture was a strong predictor of turnover intention. Organizational policies such as higher compensation and the worker’s perception that they were treated fairly tended to reduce the turnover intention of the mental health agency workforce. 

This set of studies demonstrates that workers’ intent-to-turnover is as much shaped by organizational conditions and worker characteristics as it is by the content of the work itself. Not only are workers intent-to-turnover complex and multi-faceted, but they are also likely interactional with other factors in the context of TIC, such as each worker’s personal characteristics and how they are supported by the challenging nature of their work. Therefore, it is necessary to examine linkages between staff personal characteristics and the organizational environment to more fully establish the factors that influence turnover intentions. 

### 1.4. Organizational Attachment 

Like interpersonal attachments, individuals’ psychological affective bonds toward their employing organizations [23,24] are also likely to be key factors in supporting TIC implementation. Much of the published organizational attachment research examines multilevel connections between staff and their organizations. Research in this area suggests that staff’s tenure at their organization, retirement benefits, education, age, felt participation, perceived prestige, job involvement, and role ambiguity are positively correlated with organizational attachment [25,26,27]. 

Related to organizational attachment is the concept of organizational commitment, comprised of affective, normative, and continuous elements [28,29]. This study focuses on the affective dimension, which refers to the worker’s affective sense of the organization, while normative commitment refers to needing the organization and believing that leaving it will be costly [25]. Continuous commitment refers to a staff’s sense of obligation to the organization [25,28]. Throughout the rest of this paper, “organizational attachment” will refer strictly to the affective dimension.

Organizational attachment and occupational commitment are highly correlated, suggesting a clear link between staff’s feelings toward their organization and their feelings about their chosen profession. When staff’s organizational commitment is based on shared individual and organizational values, they are more likely to express intent to remain in their position and then to actually do so [23,30]. When staff experience pride in their affiliation with their organization, they are also more likely to engage in prosocial acts (e.g., although unrequired, I perform tasks that help the organization’s image and help onboard new staff) that are not directly specified in their job description and that benefit the organization. These prosocial acts, which contribute to the mission of the organization and build relationships, are further associated with staff remaining in their positions. In contrast, organizational compliance, which occurs when workers are forced to do work that is not aligned with their personal values or with the stated values of the organization’s commitment, is significantly associated with staff intent to leave [23]. Taken together, these studies suggest a strong association between staff’s affective feelings about their organization and their intent-to-turnover in non-mental health settings. 

While several articles examined intent-to-turnover and regressed on organization attachment [30,31], others highlighted the bidirectional nature of those relationships’ dynamics [32,33]. This study examines the relationship between staff rejection sensitivity and organizational attachment among several mental health agencies transitioning to TIC. To date, no study has examined the relationship between rejection sensitivity and organizational attachment, and few studies have investigated the consequences of organizational attachment on turnover intentions. Further, the literature that has examined the latter is quite old, and it is not clear that these relationships are held in contemporary contexts. Examining the relationship between personal and organizational attachment contributes to a better understanding of factors influencing turnover intentions in mental health agencies implementing TIC. Reducing staff turnover is essential to fulfilling the promise of TIC and achieving the sustainability of adopting evidence-based TIC models. 

### 1.5. Study Aims

In the current exploratory study, we ask the following three questions: (1) Is rejection sensitivity associated with a measure of organizational attachment? (2) Do staff turnover intentions account for the unique variance in the association between rejection sensitivity and organizational attachment? Furthermore, (3) Do those associations hold once taking into account staff demographic factors (gender, race–ethnicity, education, and income)? We hypothesized that staff with higher rejection sensitivity would be less likely to express an attachment to their agency, which in turn would increase their intent to turnover and lead to staff being less committed toward implementing a new TIC intervention. We also hypothesized that staff with more organizational detachment would be more likely with intent-to-turnover within the following year. This study aims to inform policy and practice by further identifying the dynamics of turnover intentions in mental health service agencies implementing TIC. 

## 2. Method

### 2.1. Data Source

Data was collected from frontline staff, supervisors, and administrators of three mental health agencies as part of a larger study investigating the implementation of a TIC model, Attachment, Regulation and Competency (ARC). ARC is an evidence-based TIC intervention to address complex trauma. Core components of ARC include improving staff’s regulation of their own affect and establishing emotional and relational safety with clients. ARC focuses on responding effectively to clients’ affective and behavioral dysregulation as a means of reducing trauma symptoms [34]. After obtaining IRB approval, agencies that were beginning to implement ARC were enrolled in this study. Each agency joined this study at different times between 2016 and 2019. Prior to each agency’s ARC training, staff were administered an original survey measuring constructs related to implementation processes and outcomes such as job performance and satisfaction, organizational conditions and support, beliefs and attitudes about TIC, and staff relational capacities. The survey was comprised of validated measures assessing these different domains. Participants received an electronic link to the survey via email and accessed the survey using Qualtrics. This study was approved by the Institutional Review Board. 

### 2.2. Participants

The sample was drawn from 373 participants and staff from the three child and family-serving mental health agencies. Participants were excluded from the final sample if they were missing responses to any of the variables in our final analyses, resulting in 180 respondents. Descriptive results are presented in Table 1 below. 

For the purpose of this study, the race was dichotomized as White, Non-Latino, and Non-White. A score of “1” indicated Non-White staff, while a score of “0” indicated White, Non-Hispanic staff. A range of educational experiences was also recorded, with 6% of participants reporting they completed high school or their GED; 10% completing some college; 23% graduated from college; 7% received some masters training; 51% had completed their masters’ degree; and 3% completed their doctorate. Staff positions also varied in their agencies, though participants could choose all that applied, and in some cases, supervisors were also the clinical staff; thus, percentages may equal more than 100. Among staff, 45% were clinicians and program managers; 27% were program managers; and 15% of participants identified as other support staff engaged in clinical work (such as resident advisors in the residential treatment program). Consistent with the different types of positions represented in the sample, participants also reported a range of incomes. Two percent of participants reported income of less than $20,000, 33% between $20,000 and $40,000, 43% between $40,000 and $60,000, 14% between $60,000 and $80,000, and 8% made $80,000 or more. 

### 2.3. Measures 

Dependent Variable. Organizational attachment was measured utilizing the widely used Affective Commitment Scale of the Affective, Normative, and Continuance Commitment measure [35]. The Affective Commitment Scale is intended to measure an employee’s emotional attachment to an organization. This scale comprises four items utilizing a six-point scale ranging from 1 = strongly disagree to 6 = strongly agree. The measure has good internal validity, with a Cronbach’s alpha ranging from 0.77 to 0.88 [35,36]. Organizational attachment was assessed through participants’ ratings of four statements, including, “This organization has a great deal of personal meaning for me” and “I do not feel emotionally attached to this organization”. In the final analyses of 180 participants, the organizational attachment scale had a Cronbach’s alpha of 0.88. 

Independent Variables. Rejection sensitivity was measured using the Rejection Sensitivity Questionnaire, Adult version (A-RSQ). The A-RSQ is an 18-item scale that measures the cognitive–affective processes of how rejection-prone one is to situations and experiences [14]. Items include two-part questions per item. Part 1 of each question presents a scenario and asks how concerned the participant would be about the person’s reaction. Part 2 of each question asks about the participant’s expectations regarding the scenario. Items include Part 1, “You ask your parents or another family member for a loan to help you through a difficult financial time. How concerned or anxious would you be over whether or not your family would want to help you?” and Part 2, “I would expect that they would agree to help as much as they can.” Items are measured using a six-point scale. Part 1′s potential responses ranged from 1 = Very Unconcerned to 6 = Very Concerned, and for Part 2, response options ranged from 1 = Very Unlikely to 6 = Very Likely. Based on the original Rejection Sensitivity Questionnaire, the A-RSQ had high internal reliability (a = 0.83), with items loading at 0.30 or greater [14]. Among the 156 participants in our sample, the A-RSQ resulted in a Cronbach’s Alpha of 0.84. 

In addition to demographic characteristics, the final models included two other variables as covariates. The first variable was the staff turnover intention. Intent-to-turnover was measured using one item from the 3-item Turnover Intentions Scale [37]. The widely used item employed in the current analysis, “I will probably look for a new job within the next year”, has been shown to strongly correlate with the full scale [37]. The item is measured using a five-point balanced Likert-type scale (1 = Strongly Disagree, and 5 = Strongly Agree).

The second variable of interest was participants’ previous experience training in TIC, which was assessed using the question, “Have you previously been trained in TIC?” A score of “1” indicated that staff had previous training in TIC, while a score of “0” indicated that staff did not have previous TIC training. Identifying whether or not respondents had previous TIC training is necessary for understanding if their organizational attachment is partly explained by their interest in working for TIC-adopting organizations. 

### 2.4. Analytic Approach 

We conducted ordinary least squares regression analyses to investigate the association between rejection sensitivity and intent-to-turnover with organizational attachment (see Table 2). Analyses were completed using IBM SPSS version 26. 

To test whether higher rejection sensitivity is associated with lower levels of organizational attachment, layered OLS models were used to regress organizational attachment on rejection sensitivity. In Model 2, intent-to-turnover was added to the analysis by regressing organizational attachment on rejection sensitivity and intent to turnover. Model 3 added additional covariates to estimate the strength of the association and whether any significant associations could be accounted for by the addition of staff demographics to the model. Finally, the fully explicated Model 4 added previous staff trauma training to estimate whether previous trauma training would account for any of the effects in the earlier models. 

Sensitivity analyses were conducted, revealing that the sample of included participants did not differ demographically from those study participants who were excluded from this analysis. Missing data likely represents survey fatigue, as the majority of missing responses occurred during the second half of the hour-long survey.

## 3. Results

Across all four models, higher rejection sensitivity remained significantly associated with lower levels of attachment to the organization (*p* < 0.05; see Table 2). Model 1 demonstrated that higher rejection sensitivity was associated with lower organizational attachment (β = −0.39, *p* < 0.001). This bivariate model accounted for 6% of the variance in organizational attachment. With the addition of the measure of turnover intention in Model 2, intent-to-turnover was associated with lower organizational attachment (β = −0.32, *p* < 0.001), but rejection sensitivity continued to significantly predict organizational attachment (β = −0.31, *p* < 0.01). In Model 2, rejection sensitivity and turnover intention accounted for 17% of the variance in organizational attachment. 

When staff demographic covariates were layered into Model 3, higher sensitivity to rejection continued to be significantly associated with lower levels of organizational attachment (β = −0.26, *p* < 0.05), as was intent-to-turnover (β = −0.29, *p* < 0.001). Moreover, the addition of staff demographics showed that the more education one has, the less attached they feel to the organization (β = −0.15, *p* < 0.05). Model 3 accounted for 22% of the variance in organizational attachment. 

Finally, with the addition of the measure of previous trauma training in Model 4, higher rejection sensitivity maintained its significant negative association with organizational attachment (β = −0.27, *p* < 0.05), with 22% of the variance explained. Intent-to-turnover also continued to be a strong indicator of lower organizational attachment (β = −0.29, *p* < 0.001), as did more education (β = −0.15, *p* < 0.05) in this fully layered model.

## 4. Discussion

This study is the first to investigate the relationship between rejection sensitivity, turnover intention, and organizational attachment. In doing so, we asked three questions: Does rejection sensitivity predict organizational attachment? Could staff turnover intentions account for the association between rejection sensitivity and organizational attachment? Moreover, once established, do these associations hold when we account for staff demographic factors? We hypothesized that staff with higher rejection sensitivities would be less likely to express an attachment to their agency, which in turn would increase their intent to turnover. This study aims to connect relational concepts with organizational factors to inform implementation science in general and the implementation of TIC in particular. Findings suggest a significant inverse association between rejection sensitivity and organizational attachment, supporting a dynamic conceptualization of how personal and organizational constructs interact with one another.

The findings reveal that adults with higher rejection sensitivity may find less personal meaning and emotional attachment to the workplace. This finding builds on previous research on the personal consequences of a higher sensitivity to rejection, which identified how adults with a higher sensitivity to rejection attempt to maintain connections and prevent rejection through highly regulated adaptation efforts. These regulated adaptation efforts can take two forms: anxiety, which triggers pre-emptive withdrawal from the relationship from which one fears rejection, and/or anger, which prompts aggressive responses to ambiguous social interactions or environments [13,38].

While working in a child and family-serving mental health agency and with clients with trauma histories can be very challenging, there are significant rewards to engaging in this work. Developing meaningful professional–client relationships and observing clients’ resilience and empowerment as they heal is often very fulfilling [39,40]. For staff with a greater sensitivity to rejection, these rewards may be harder to access. Maintaining workplace interrelationships between colleagues and clients could be more stressful, and accessing the relational rewards of this feeling may be more demanding. Specifically, higher rejection sensitivity may contribute to meaning loss and emotional disengagement in both staff–client and staff–workplace relationships, which may lead to organizational detachment.

Organizational attachment in mental health service settings that treat complex trauma may be an important and overlooked component of strengthening TIC implementation, where strong working relationships and trust are critical staff qualities [41] in general and a requirement for the provision of TIC in particular. For staff with personal histories of unresolved loss and trauma who have a higher sensitivity to rejection, their defensive motivational system may impinge on their ability to develop strong organizational attachment, which in turn leads to intent to turnover. Staff expressing greater turnover intention were significantly less strongly attached to their organization (β = −0.29, *p* < 0.001). This finding supports the hypothesis that adults with higher rejection sensitivity tend to express more turnover intention and are less attached to the organization. Staff with higher levels of rejection sensitivity may need more space and support to integrate their relational styles with the demands of TIC.

While workplace challenges related to structural or organizational features are often primarily considered the reason for implementation challenges, these findings suggest that relational and personal characteristics dynamically interact with the work environment to shape constructs like organizational attachment and intent to turnover. In short, these are likely to be nested and transactional factors rather than separate components. Our finding indicates that those with a higher rejection sensitivity are more likely to have a lower organizational attachment and are more likely to express an intent to turnover. This finding is supported by other research findings that link personal, relational, and organizational constructs. Specifically, Scrima et al. (2015), whose work demonstrates that avoidant attachment styles are associated both with higher turnover intentions and lower organizational commitment levels (p. 432, [42]).

Of the examined staff demographic factors, only higher levels of education significantly predicted lower organizational attachment (β = −0.15, *p* < 0.05). While earlier studies did not explicitly examine rejection sensitivity, educational level, and organizational attachment interrelationships, this finding aligns with organizational commitment literature, where staff with higher educational levels are likely to express greater leave intentions attributed to available work alternatives. These results may point to the difficulty staff face in finding meaning and emotional connection to the workplace. Child and family-serving mental health agency staff often struggle between their professional mandate to provide consistent and relational care and well-documented structural challenges in providing mental health services [43,44,45]. Weaver et al. (2007) found that, unlike other degree holders, “staff with [Master in Social Work] MSWs are more likely to express intentions to leave the job” (p. 21, [46]), which may speak to the pervasive challenges organizations face in social service and mental health settings, namely time constraints, countervailing pressures to surveil and support clients, administrative and financial burdens, and complex client symptoms that are amplified by structural inequality and environmental constraints. The finding that higher degree holders were more likely to express an intent-to-turnover likely reflects the nuanced terrain of this landscape.

More training in TIC is unlikely to increase organizational attachment and decrease intent-to-turnover for those who struggle with a higher sensitivity to rejection. Layering in previous trauma training into the model did not add to the variance in organizational attachment accounted for. In this model, turnover intention also continued to be a strong indicator of lower organizational attachment, as did higher staff education. Results suggest that personal and relational constructs shaped by histories of loss and trauma are unlikely to be mitigated simply by educational approaches. This finding is critical, especially as the number of child and family-serving mental health agencies transitioning to become TIC-based organizations is growing rapidly, and many are focused on training their staff in this new framework for care. In parallel to the principles of TIC, relationally centered approaches are likely critical to building organizational attachment. This is especially true for staff who may struggle with their own feelings of relational safety, which likely translates to their feelings about their organization.

These findings add to the growing literature suggesting that implementation of TIC requires a focus on staff histories of loss and trauma [9,21] through supervisory support. The study by Collin-Vézina et al. (2020) revealed that compared to frontline staff, managers scored higher on worker self-efficacy, response to problem behavior, and on-the-job behavior measures, and workers with a community college degree—and not a university degree—indicated a greater sense of self-efficacy. Consequently, we recommend intensive TIC-focused training to enhance frontline staff’s knowledge, self-efficacy, capacity, and support in recognizing and understanding clients’ trauma and possibly mitigate vicarious traumatization. Child and family-serving mental health agencies may benefit from being curious about staff’s feelings of organizational attachment and intentions or desires to remain or stay with the organization. Opportunities to build a sense of community, belonging, and shared purpose will likely support staff in finding meaning in their work and informing an emotional connection to their organization. Prior research suggests that organizational attachment can be enhanced by focusing on different elements of staff commitment [47]. Organizations that nurture and support staff’s affective commitment can likely decrease turnover. In the context of transitions to TIC, increasing organizational attachment and decreasing turnover have the potential to support the implementation and sustainment of TIC models. One important aspect of this is ensuring that staff have a space to process how their own relational styles and histories of loss and trauma influence their approach to the work.

Child and family-serving mental health agencies should also adopt a sensitive approach that attends to the on-the-ground challenges that shift staff intentions to turnover into actual departures. Organizations should recognize that not all staff might be a good fit for the emotional demands of TIC. In these cases, child and family-serving mental health agencies transitioning to TIC should offer their staff who wish to leave a smooth pathway for a transition in which they feel supported in their next steps and valued for the contributions that they have already made. Such a process will ensure that those staff who find staying emotionally taxing do not feel they must remain in a job that is no longer a good fit, while simultaneously hiring new staff who are more comfortable engaging in the emotional demands of TIC provision.

### 4.1. Limitation

This study, while the first to look at the relationship between staff members’ organizational attachment and their rejection sensitivity and intent to turnover, has several limitations to consider when interpreting its findings. A primary limitation of this study is that it was cross-sectional in nature. This design does not allow causal inference, limiting the discussion of the directionality of effects. The current study found associations between rejection sensitivity, organizational attachment, and turnover intentions. Organizational attachment is significantly negatively associated with rejection sensitivity and turnover intentions. Longitudinal analyses would be an important next step to allow for a fuller elucidation of the temporal nature and directionality of these associations.

Another limitation pertains to the restricted generalizability of the current findings because of the sample characteristics. As described in the method section, this study draws from a diverse sample and provides a unique examination of the organization–staff outcomes, yet our findings should be replicated across other social service practice domains and work settings, which may vary in important ways.

Despite the abovementioned limitations, in analyzing the hypothesized link between staff rejection sensitivity and organizational attachment orientations and related differences in turnover intentions, our study contributes to the literature in important ways: First, except for the above-cited studies, works on possible contributions of rejection sensitivity to the understanding of workplace attitudes and behaviors are scarce [48]. Thus, on a broader theoretical level and in addition to the few existing works, our research provides further evidence for extending attachment theory to the workplace domain. Second, to our knowledge, it is the first to explore the impact of rejection sensitivity orientation on employees’ affective organizational attachment in TIC transitioning mental health agencies. As outlined before, we deem the hypothesis plausible from a theoretical stance and consider it worthy of future research.

### 4.2. Implications

While cross-sectional and a first exploratory look at the connection between rejection sensitivity and organizational constructs, this study is suggestive that staff relational histories of unresolved loss and trauma expressed through their ongoing degree of sensitivity to rejection may influence their organizational attachment in a manner that accounts for unique variance in organizational attachment over and above the extent to which they intend to turnover from their job. At the very least, organizations implementing TIC would likely benefit from working to strengthen staff’s emotional connections to their organization and supporting them in understanding their own reactions and sensitivity to client behavior. Staff with a higher rejection sensitivity likely need explicit indicators of shared personal and organizational values as well as their value to the organization itself. Direct relational support from organizations and shared meaning-making about the day-to-day work are likely important for fostering trust and buying into the principles of TIC.

Child and family-serving mental health agencies endure high staff turnover rates. While some turnover rates should be acceptable to bring new energy and skills to these organizations, targeted efforts to decrease staff turnover are critical for sustaining TIC interventions and providing services founded upon principles of relational safety. Understanding that rejection sensitivity affects staff organizational attachment and turnover intentions points to the need for managers and supervisors to be curious about their staff’s experiences and set up supportive procedures like high-quality supervision. These conversations have the potential to reveal earlier signs of workplace withdrawal, especially for staff who may not intend to leave the organization but who have withdrawn from the work itself. Organizational support that is relationally based will likely help staff who struggle with rejection sensitivity develop their relational capacities.

We see this work as underscoring the need for increased research attention and longitudinal analyses to more fully determine if staff with higher rejection sensitivity may benefit from explicit training and supervision supports to understand and modulate their bidirectional processes with client behavior perceived as threatening and indicators of shared personal and organizational values as well as their value to the organization itself.

Our results pose a cautionary and inevitable practical question: whether mental health agencies should avoid recruiting staff with higher rejection sensitivity. We do not feel our data supports such a conclusion. Pathologizing staff who have a higher sensitivity to rejection would not be in keeping with the principles of TIC, nor would it be ethical. Instead, this study emphasizes the reality of the workforce, where professionals in mental health are more likely to report higher rates of their own traumatic exposure and adverse childhood experiences [49]. To respond to these realities and their potential practice implications, we recommend supportive measures at the organizational level. Procedures for staff to receive peer support after particularly challenging client interactions or a break after dealing with a crisis are examples of organizational strategies that could enhance affective commitment among all staff and which may attend to the needs of staff who have higher rejection sensitivities. Also, training and supervision help staff understand their own emotional reactions to client interactions perceived as threatening and have the potential to disarm and reframe the reactivity. Considering the role of staff relational capacities in shaping staff–staff and client–staff relationships is important for organizations to wrestle with as they implement TIC.

Relational leadership that promotes mutual trust and collaboration and attends to staff’s experiences within the organization is likely to lead to higher job satisfaction and lower job turnover by promoting organizational attachment (p. 2, [50]). Organizational strategies that recognize there is a parallel process between staff experiences within their work environment and how staff approach clients are also critical. Relational leadership necessarily includes regular opportunities for staff to work through conflict and consider organizational functioning [51]. Such opportunities, facilitated by relational leadership, enable building and sustaining organizational attachment. One benefit of these strategies is that they address various causes of diminished organizational attachment. One benefit of these strategies is that they may address various causes of diminished organizational attachment, and future TIC implementation science should serve to elucidate these complex multi-directional relational processes more fully.

## 5. Conclusions

This study builds on an emerging body of research that seeks to connect staff personal histories of loss and trauma to organizational constructs such as organizational attachment and intent to turnover. We find that staff with a higher sensitivity to rejection are more likely to express a lower level of organizational attachment, even taking into account their intent to leave their organization. Relational safety is foundational to the effective implementation of TIC, which is undermined by frequent staff turnover. Providing organizational support to staff may be one way to increase organizational attachment and reduce turnover.

## Figures and Tables

**Table 1:** Study sample descriptives.

n = 180	Mean or % (n)	SD	Min	Max
Organizational Attachment	4.11	1.13	1	7
Rejection Sensitivity	2.62	0.72	1	5
Intent to Turnover	2.23	1.2	1	5
Sex				
Male	16% (29)			
Female	84% (151)			
Race				
Non-White	43%(77)			
White	57% (103)			
Hispanic				
Hispanic	21% (37)			
Not Hispanic	79% (143)			
Education Level				
Completed HS or GED	6% (11)			
Some College	10% (18)			
Completed College	23% (42)			
Some Masters Completed	7% (12)			
Masters Completed	51% (91)			
Completed Ph.D. or equivalent	3% (6)			
Staff Position *				
Clinician	45% (81)			
Program Manager	27% (48)			
Residential Associate	14% (26)			
Child Care Worker	2% (4)			
Supervisor	1% (2)			
Case Manager	1% (2)			
Other Support Staff	15% (27)			
Annual Income				
<$20,000	2% (4)			
$20,000–$40,000	33% (59)			
$40,000–$60,000	43% (78)			
$60,000–$80,000	14% (25)			
$80,000+	8% (14)			
Trauma Knowledge				
Prior Trauma Training	54% (97)			
No Prior Trauma Training	46% (83)			

* Staff could choose all that apply descriptive statistics.

**Table 2:** The standardized coefficient associated with OLS regression for all hypotheses.

		Organizational Attachment		
n = 180	Model 1	Model 2	Model 3	Model 4
Rejection Sensitivity	−0.39 ***	−0.31 **	−0.26 *	−0.27 *
Intent to Turnover Female ^1^		−0.32 ***	−0.29 *** 0.29	−0.29 *** 0.28
POC ^2^			−0.10	−0.10
Hispanic ^3^			−0.06	−0.06
Education Level			−0.15 *	−0.15 *
Annual Income ^4^			0.1	0.09
Prior Trauma Training				−0.06
*r* ^2^	0.06	0.17	0.22	0.22

*** *p* < 0.001 ** *p* < 0.01 * *p* < 0.05. ^1^ Reference group is male. ^2^ Reference group is White. ^3^ Reference group is not Hispanic/Latino. ^4^ Increments of 20,000.

## Data Availability

The deidentified data presented in this study are available on request from the corresponding author.
